# Effects of *N*-acetylcysteine on CG8005 gene-mediated proliferation and apoptosis of *Drosophila* S2 embryonic cells

**DOI:** 10.1038/s41598-023-39668-4

**Published:** 2023-08-02

**Authors:** Wanyin Chen, Yifei Yin, Zheng Zhang

**Affiliations:** 1 Department of Medical Gynecology, Affiliated Hospital of Jiangsu University, Zhenjiang, 212000 People’s Republic of China; 2 Department of Medical Ultrasound, Affiliated Hospital of Nantong University, Nantong, 226006 People’s Republic of China; 3 Department of Medical Ultrasound, Affiliated Hospital of Jiangsu University, Zhenjiang, 212000 People’s Republic of China

**Keywords:** Genetics, Genetic markers

## Abstract

To investigate the effect of the antioxidant *N*-acetylcysteine (NAC) on the proliferation and apoptosis in CG8005 gene-interfering *Drosophila* S2 embryonic cells by scavenging intracellular reactive oxygen species (ROS). The interfering efficiency of CG8005 gene in *Drosophila* S2 embryonic cells was verified by real-time quantitative PCR (qRT-PCR). Different concentrations of NAC and phosphate buffered saline (PBS) were used to affect the *Drosophila* S2 embryonic cells. The growth state of *Drosophila* S2 embryonic cells was observed by light microscope. Two probes dihydroethidium (DHE) and 2,7-dichlorodihydrofluorescein-acetoacetate (DCFH-DA) were used to observe the ROS production in each group after immunofluorescence staining. TUNEL staining and flow cytometry were used to investigate the apoptosis level of *Drosophila* S2 embryos, and CCK-8 (Cell Counting Kit-8) was used to detect the cell viability of *Drosophila* S2 embryos. The knockdown efficiency of siCG8005-2 fragment was high and stable, which was verified by interference efficiency (*P* < 0.05). There was no significant change in the growth of *Drosophila* S2 embryonic cells after the treatment of NAC as compared to PBS group. Moreover, knockdowning CG8005 gene resulted in an increase in ROS and apoptosis in *Drosophila* S2 embryonic cells (*P* < 0.05) and a decrease in proliferation activity (*P* < 0.05). In addition, the pretreatment of antioxidant NAC could inhibit ROS production in *Drosophila* S2 embryonic cells (*P* < 0.05), reduce cell apoptosis (*P* < 0.05), and improve cell survival (*P* < 0.05). The CG8005 gene in *Drosophila* S2 embryonic cells could regulate the proliferation and apoptosis of S2 embryonic cells by disrupting the redox homeostasis, and antioxidant NAC could inhibit cell apoptosis and promotes cell proliferation by scavenging ROS in *Drosophila* S2 embryonic cells, which is expected to provide novel insights for the pathogenesis of male infertility and spermatogenesis.

## Introduction

Intracellular redox homeostasis, which refers to the dynamic balance between oxidizing and reducing species, plays a critical role in maintaining normal physiological processes, including cell growth, metabolism, apoptosis and proliferation^1,2^. When the body is subjected to harmful stimuli, the oxidative system and the antioxidant system are out of balance and then tend to be oxidative, thereby resulting in neutrophil infiltration, protease secretion, and a large number of oxidative intermediates production^3,4^. Reactive oxygen species (ROS) is a general term for active oxygen-containing compounds including hydroxyl radical (·OH), hydrogen peroxide (H_2_O_2_) and superoxide radical (O_2_^−^), which was generated in the process of oxidative metabolism^5–7^. Under the normal condition, the body can produce a small amount of ROS to maintain the balance of oxygen metabolism under the effects of free radical scavenging enzymes and antioxidants^8,9^. However, a large amount of ROS can be produced after the harmful stimuli, which causes the disruption of the redox homeostasis, and ultimately contributing to the occurrence of oxidative stress^10^. During recent years, accumulating evidence has shown that oxidative stress is closely involved in the occurrence of cell proliferation and apoptosis. The treatment of oxidants can induce apoptosis and inhibit cell proliferation, while the use of antioxidants can reduce apoptosis and increase cell viability^11,12^. *N*-acetylcysteine (NAC), a thiol- containing antioxidant and glutathione (GSH) precursor, could attenuates oxidative stress by scavenging free radicals and stimulating antioxidant enzyme activity^13,14^. There is a report that the use of NAC has also been associated with a significant reduction in lipid peroxidation and a significant increase in GSH levels in the liver and erythrocytes of mice^15^.

CG8005 is a deoxygenated Threonine synthase that catalyzes NAD dependent oxidative cleavage of Spermidine. Using Bing (string db. Org/network/7227.FBpp0072081) online tool analysis, there may be interaction between Initiation factor 5A (elf-5A) protein and Eukaryotic translation, which may be involved in oxidative stress response and cell membrane integrity^16^. Previous studies have shown that CG8005 can affect oxidative stress response, and participate in the proliferation and apoptosis process of *Drosophila* testicular reproductive stem cells^17^.

*Drosophila* S2 embryonic cells, as a cell model for expressing foreign proteins, are superior to common mammalian cells. When the target gene is integrated into the cell genome through experimental operations, *Drosophila* S2 embryonic cells can complete the correct transcription, translation and protein processing. And the target protein is structurally and functionally identical to the natural protein^18^. At the same time, *Drosophila* S2 embryonic cells are semi-suspended cells, which are possessed with the advantages of convenient culture and rapid proliferation. In our study, *Drosophila* S2 embryonic cells were functioned as an in vitro model to detect the oxidative level by regulating the expression of the CG8005 gene, and the effect of NAC on the proliferation and apoptosis in *Drosophila* S2 embryonic cells was also investigated in our study^19^.

## Materials and methods

### Cell lines

*Drosophila* S2 embryonic cells were obtained from *Drosophila* Genomics Resource Center.

### Reagents and instruments

The reagents and equipment information used in this study are as follows: DMEM (Gibco, USA), Fetal Bovine Serum (Bioind, Israel), LipofectamineTM2000 (Invitrogen, USA), opti-MEM (Gibco, USA), Small Interfering RNA (Suzhou Gema Gene), *N*-acetyl-l-cysteine (Shanghai Biyuntian), TRIzol reagent (TaKaRa, Japan), Prime Script RT Reagent Ki reverse transcription instructions (TaKaRa, Japan), SYBG master mix dye (TaKaRa, Japan), 2,7-dichlorodihydrogen Fluorescein-acetoacetate (DCFH-DA) and 1uM dihydroethidium (DHE) (Shanghai Biyuntian Biotechnology Co., Ltd.), DAPI (Invitrogen, USA). Temperature incubator (Shanghai Jinghong Experimental Equipment Co., Ltd.), Mx3000P fluorescence quantitative PCR instrument (Agilent, USA).

### Methods

#### Cell culture and transfection

*Drosophila* S2 embryonic cells frozen in − 80 °C liquid nitrogen tank were resuscitated following the principle of slow freezing and fast thawing. A complete culture medium was prepared based on DMEM (Gibco, USA) + 10% fetal bovine serum (Bioind, Israel). *Drosophila* S2 embryonic cells were cultivated at 28 °C constant temperature under CO_2_ free atmosphere. Cells were cultured in an appropriate density and inoculated into a new culture bottle for further cultivation and subsequent experiments.

*Drosophila* S2 embryonic cells were inoculated onto a 6-well plate in the density of 1.5 × 10^5^ cells/ml for the cell transfection according to the instruction of the liposome LipofectamineTM2000 (Invitrogen, USA) transfection reagent. 15 ul small interfering RNA (siRNA designed and synthesized by Suzhou Jima Gene Company) was added to 250 ul of optim MEM, and 15 ul LipofectamineTM2000 was added into 250 ul optim MEM. These two solutions were stood for another 5 min at the room temperature. Subsequently, two mixed solutions were fully fused, and then stood for another 20 min at the room temperature. Finally, a concentration of 150 nmol/l mixed solution was added into a 6-well plate for cell transfection. After 6 h transfection, and the new culture medium was replaced for continued incubation for another 48 h in a constant temperature incubator. siRNA information was listed in Table 1.

**Table 1:** siRNA sequences.

siRNA name	Sequence (5′-3′)
Negative control	F: UUCUCCG AACGUGUCACGUTT
	R: ACGUGACACGUUCGGAGAATT
siCG8005-1	F: GGACCAAAUAGACAGCCAUTT
	R: AUGGCUGUCUAUUUGGUCCTT
siCG8005-2	F: CCACGUUCAUGGGUUCAUUTT
	R: AAUGAACCCAUGAACGUGGTT

#### NAC intervention

S2 cells of *Drosophila* melanogaster were inoculated into a 6-well plate in a cell density of 1.5 × 10^5^ cells/ml. 1.25 mM NAC solution, 2.5 mM NAC solution, 5.0 mM NAC solution, 3.75 ul PBS solution, 7.5 ul PBS solution, and 15 ul PBS solution were added to each well for pre-treatment, respectively. S2 cells were co-incubated for 1 h to change the intracellular ROS level. Then, Negative Control and 150 nmol/L siCG8005 transfection solution (prepared according to step 1.3.1, with a final volume of 530 ul) were added to the corresponding six well plate. S2 cells were co-incubated in a constant temperature incubator and completed a series of experimental operations.

#### Real-time fluorescence quantitative PCR detection

Total RNA was extracted using TRIzol reagent (TaKaRa, Japan). RNA concentration was measured using UV spectrophotometer, and transcribe RNA was reversed into cDNA according to Prime Script RT Reagent Ki reverse transcription instructions (TaKaRa, Japan). SYBG master mix dye (TaKaRa, Japan) was prepared with cDNA and corresponding primers to form a reaction system (ice operation), which was fully mixed and briefly centrifuged. The Mx3000P fluorescence quantitative PCR instrument (Agilent, USA) was utilized for the subsequent reaction. Glyceraldehyde-3-phosphate dehydrogenase (GAPDH) was used as an internal reference gene. According to the manufacturer’s regulations, the standard curve was used to calculate the multiple relationship with Folds = 2^ − ΔΔ^ CT representing the ploidy relationship between the expression of the target gene in the experimental group and the control group. Each experiment was repeated three times independently. (Table 2).

**Table 2:** qRT-PCR primer sequences.

siRNA name	Sequence (5′-3′)
GAPDH	F: GTGGTGAACGGCCAGAAGAT
	R: GCCTTGTCAATGGTGGTGAA
CG8005	F: CTGGACTCGTGGTGGACATTCTG
	R: ACACTGAGTAATCCGCTCCATTGC

All primers were synthesized by Shanghai Sangon Industrial Co., Ltd.

#### ROS detection

ROS assay kit is the commonly used method for the detection of ROS generation based on the change of fluorescence intensity of fluorescent dye DCFH -DA (2,7 -dichlorofluorescein diacetate). Intracellular ROS can oxidize non-fluorescent DCFH to generate fluorescent DCF (dichlorofluorescein). After corresponding treatment for 48 h, the S2 cells were plated into 96-well plates and incubated with 10 μM DCF for 30 min at 37 °C. After the DCF was removed, the cells were washed with PBS, and the fluorescence of the cells from each well was measured by a SYNERGY microplate reader. Meanwhile, the cells plated into 24-well plates were photographed, and fluorescence intensity analysis was performed as described for DHE staining.

#### TUNEL staining

Cell apoptosis was determined using the TUNEL assay according to the manufacturer’s protocols. The TUNEL BrightRed Apoptosis Detection Kit was obtained from Vazyme. The marking solution according to the instructions in Table 3.

**Table 3:** Configuration of marking solution.

5 × Equilibration buffer	10 ul
BrightRed labeling mix	5 ul
Recombination TdT enzyme	1 ul
ddH_2_O	34 ul

#### CCK-8 cell viability detection

The cell viability of *Drosophila* S2 embryonic cells was detected using CCK-8 (cell counting kit-8) assay. *Drosophila* S2 embryonic cells were incubated in 96-well plates (10^4^ cells per well) for 12 h. Pre-prepared 10% CCK-8 culture medium was added to each well and then co-incubated for another 1 h in a 37 °C incubator. The absorbance at 450 nm was measured using an enzyme-linked immunosorbent assay after transfection for 0, 24, and 48 h. The O.D values were recorded and plotted using GraphPad software.

#### Flow cytometry

Annexin V and propidium iodide (PI) dyes were used as fluorescent probes to detect the apoptosis by flow cytometry. *Drosophila* S2 embryonic cells were transfected according to the above steps. After transfection 48 h, the cells were washed with ice-cold PBS at 4 °C. Then, the concentration of cells was adjusted to 1 × 10^6^ cells per well based on the cell counting formula. 5 μl of Annexin V-Alexa Fluor 647 and 10 μl of PI solution were added into each sample tube, and then co-incubated at room temperature for 15 min in the dark. 200ul PBS was added to each tube of samples and then these samples were analyzed by FACS flow cytometer. The results were further organized and plotted using Flowjo software.

### Statistical methods

All quantitative data was expressed as mean ± standard deviation (s.d.) of at least three independent sample tests. Statistical comparisons were conducted by Student’s t test. Differences were considered significant at *P* < 0.05 (*P < 0.05, **P < 0.01, and ***P < 0.001).

## Results

### The interference efficiency of CG8005

**Figure 1:**
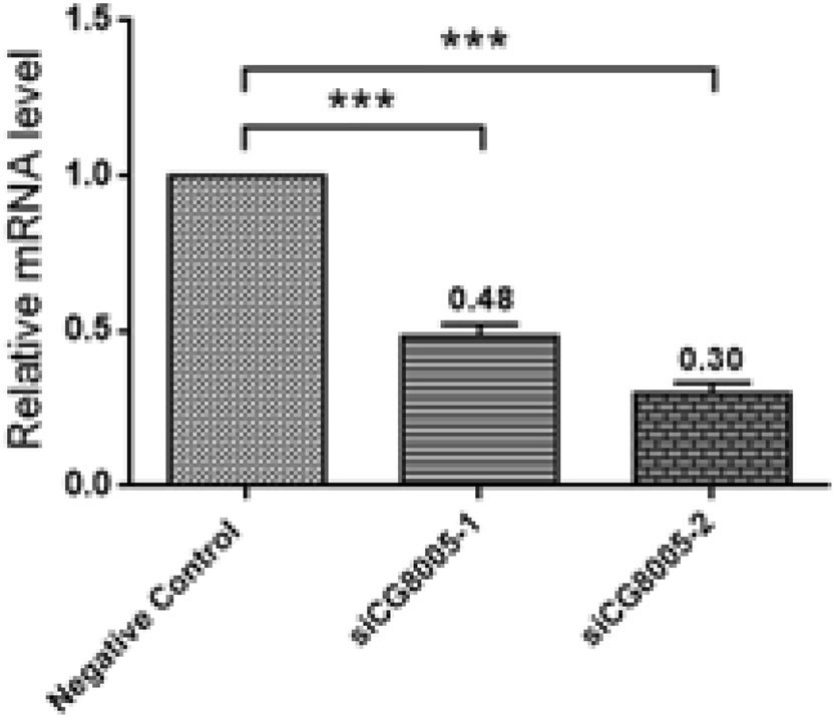
Relative CG8005 mRNA level in control and CG8005 siRNA (CG8005 siRNA-1 and CG8005siRNA-2) cells to validate knockdown efficiency. The error bars represent mean ± standard deviation (s.d.) from three replicates.

Two small interfering RNAs (siCG8005-1 and siCG8005-2) were selected to silence the CG8005 gene in *Drosophila* S2 embryonic cells. The real-time quantitative PCR was used to compare the expression level of messenger RNA (mRNA) in the negative control group, siCG8005-1 group and siCG8005-2 group. As shown in Fig. 1, the interference efficiency of the siCG8005-2 fragment was higher than that of the siCG8005-1 fragment (Fig. 1, *P* < 0.001).

### The effect of NAC in of *Drosophila* S2 embryonic cells

**Figure 2:**
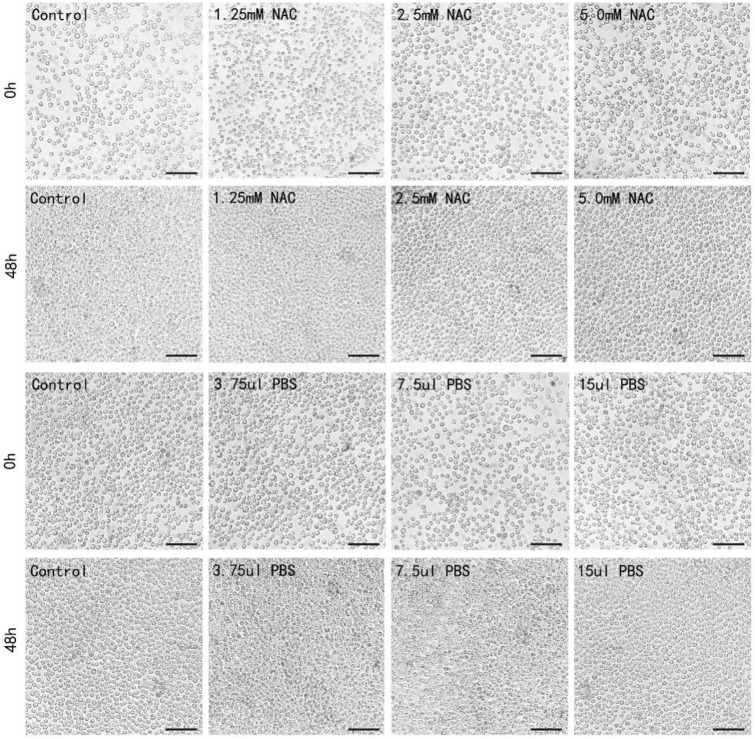
Observation of the growth of *Drosophila* S2 embryonic cells after NAC and PBS treatment. Scale bar: 30 μm.

Phosphate buffered saline (PBS) is generally used as a solvent to dissolve the protective reagent. After the *Drosophila* S2 embryonic cells were treated with different doses of PBS (3.75 ul, 7.5ul, 15 ul), the number of *Drosophila* S2 embryonic cells exponentially increased. The morphology of *Drosophila* S2 embryonic cells remained small and round, which were half-suspended in the culture bottle. Reactive oxygen scavenger *N*-acetylcysteine (NAC) is a commonly used antioxidant, which could inhibit intracellular ROS production^20^. After 48 h of treatment with different concentrations of NAC (1.25 mM, 2.5 mM, 5.0 mM), the number of *Drosophila* S2 embryonic cells was significantly increased, while the growth state of cells was not obviously affected as compared to PBS group (Fig. 2).

### ROS level in of *Drosophila* S2 embryonic cells

**Figure 3:**
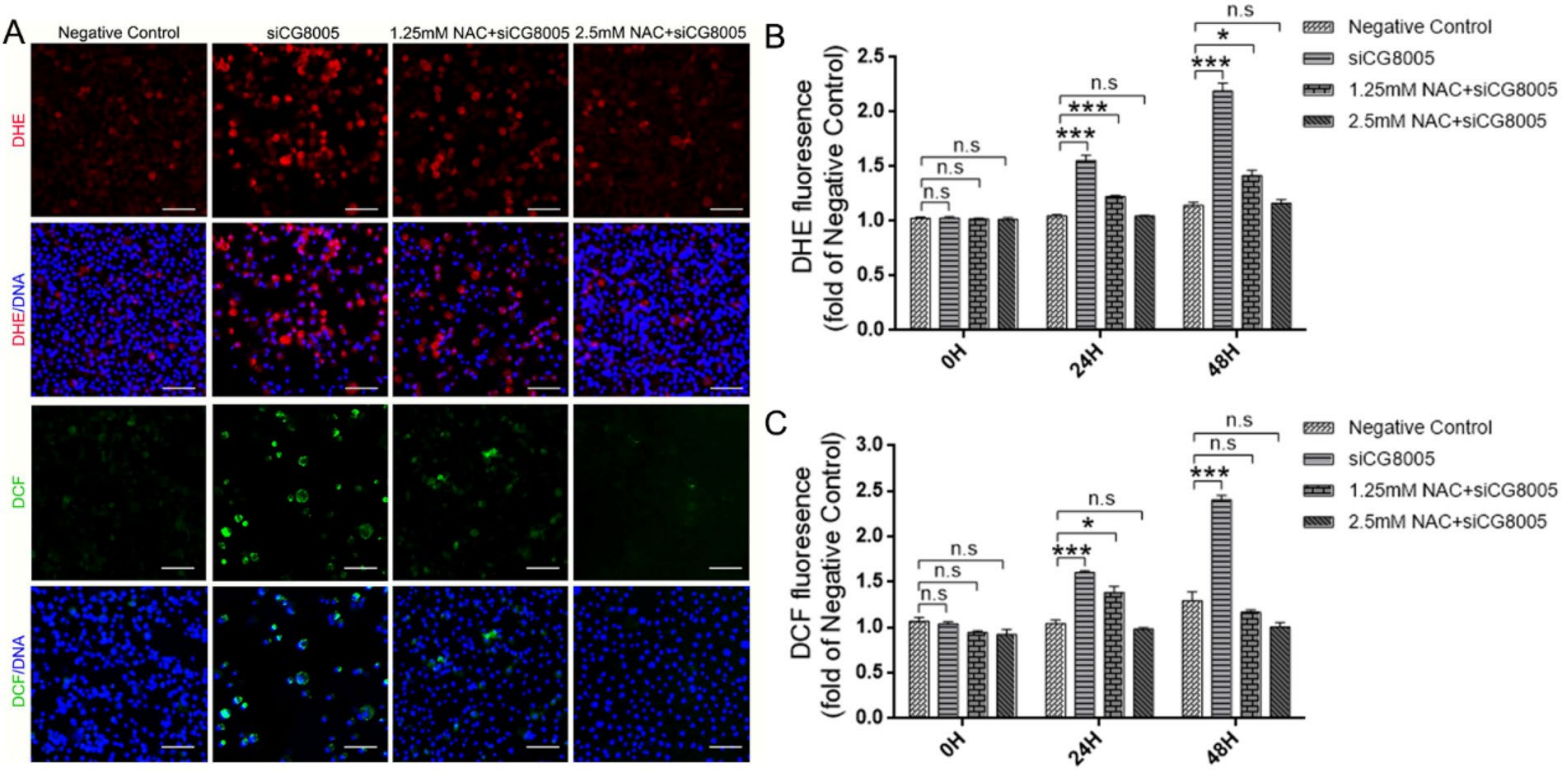
Detection of ROS content in *Drosophila* S2 embryonic cells. (**A**) Fluorescence diagram of DHE and DCF-DA in S2 cells after different treatments. Scale bar: 30 μm. (**B**) Quantitative analysis of DHE staining in S2 cells after different treatments. (**C**) Quantitative analysis of DCF staining in S2 cells after different treatments. The error bars represent mean ± standard deviation (s.d.) from three replicates in (**B**, **C**).

ROS is recognized to be essential stem cell regulator, which can influence the homeostasis of stem cell by promoting the differentiation and self-renewal in different populations of stem cell^21^. To examine whether dysfunctional CG8005-induced spermatogonial differentiation defects was related to oxidative stress, the redox state was investigated in the group of Negative Control, siCG8005 and NAC + siCG8005. After treated with siCG8005, the ROS level in S2 embryonic cells was dramatically increased. However, ROS generation in the group of NAC + siCG8005, as observed by DHE and DCF probes, was greatly decreased in a dosage-dependent effect (Fig. 3A-C).

### The apoptosis level of *Drosophila* S2 embryonic cells

**Figure 4:**
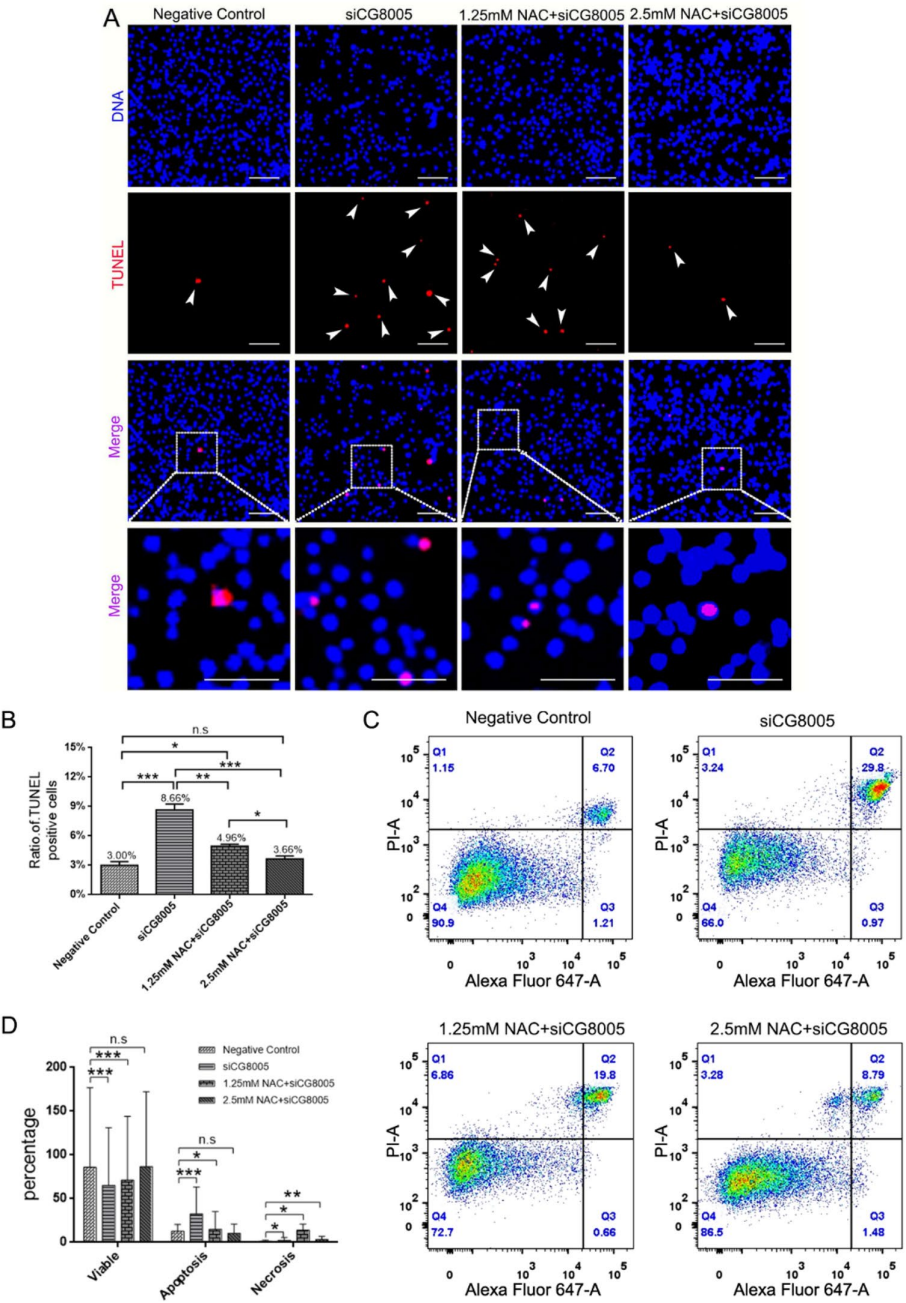
Detection of apoptosis in *Drosophila* S2 embryonic cells after different treatments. (**A**) TUNEL fluorescence in S2 cells after the siCG8005 and NAC intervention. Scale bar: 30 μm. (**B**) Quantitative analysis of TUNEL staining after different treatments. (**C**) Flow cytometry analysis of S2 cells after different treatments. (**D**) Quantitative analysis of flow cytometry apoptosis after different treatments. The error bars represent mean ± standard deviation (s.d.) from three replicates in (**B**, **D**).

To further investigate the effect of NAC on CG8005-mediated apoptosis in *Drosophila* S2 embryonic cells, TUNEL reagent was used to detect the cell apoptosis. The immunofluorescence results confirmed that the TUNEL-positive signals were significantly increased at the activation of oxidative stress. The cell apoptosis could be gradually rescued as the concentration of NAC gradually increases (Fig. 4A), which was consistent with the quantitative analysis (Fig. 4B). Flow cytometry analysis demonstrated that after knock-downing CG8005, there was a significant increase in early and late apoptotic cells as compared to the negative group. But these apoptotic cells were gradually decreased, after the treatment of NAC intervention (Fig. 4C,D).

### The proliferation level of *Drosophila* S2 embryonic cells

**Figure 5:**
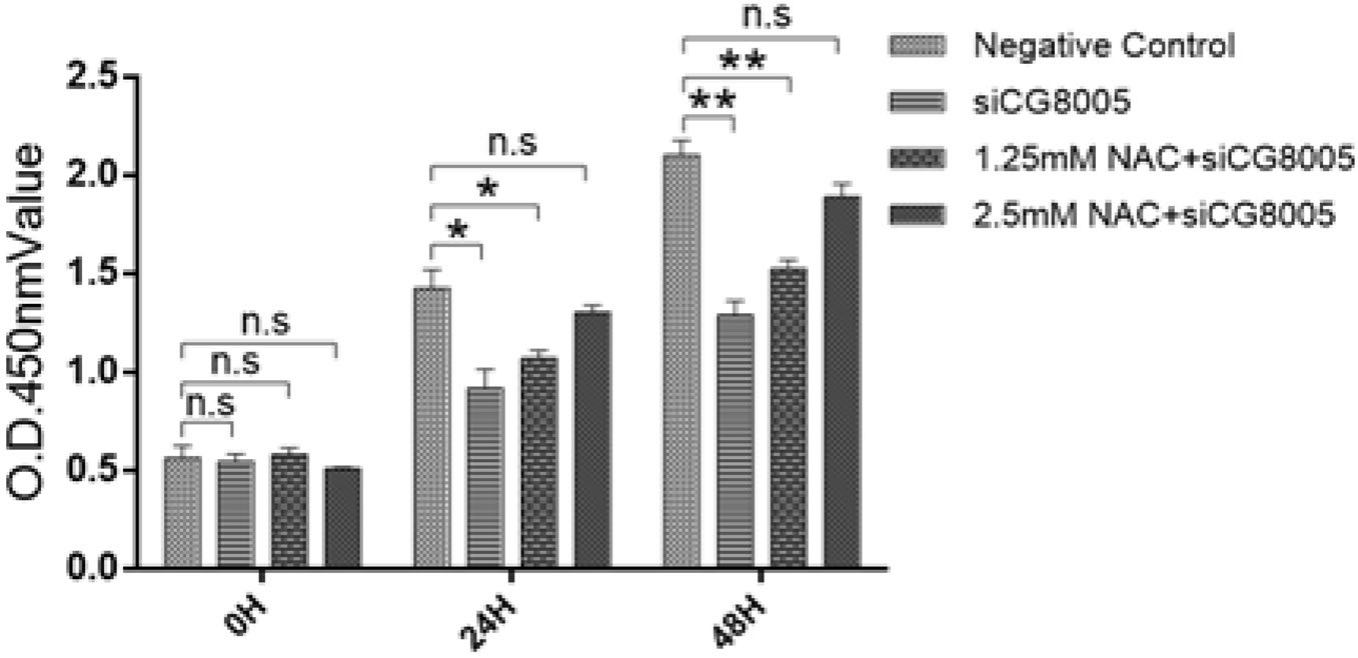
Proliferation detection of *Drosophila* S2 embryonic cells after the different treatments at the given time intervals. The error bars represent mean ± standard deviation (s.d.) from three replicates.

The proliferation activity of S2 embryonic cells after different treatments was detected using CCK-8 experiment at different time points. The proliferation activity of cells in the siCG8005 group was inhibited as compared to negative control group, which displayed a time-dependent effect. In addition, the cell inhibition of S2 embryonic cells was gradually rescued, when the concentration of NAC increased. It is noted that there was no statistical difference of cell proliferation level between the Negative Control group and the siCG8005 plus NAC group, which clearly demonstrated that antioxidant NAC inhibits cell apoptosis and promotes cell proliferation by scavenging ROS in *Drosophila* S2 embryonic cells. (Fig. 5).

## Discussion

Oxidative stress is closed associated with different kinds of human diseases, including cardiovascular diseases, neurodegenerative diseases, type II diabetes, and malignant tumors^22,23^. The disruption of redox homeostasis affects the proliferation, differentiation, and division in different stem cell populations^24^. *Drosophila* is reported to be a classic in vivo model in the field of reproductive system research, and is often used to investigate male infertility and spermatogenesis^25,26^. When *Drosophila* S2 embryonic cells are subjected to harmful stimuli, a large amount of reactive oxygen species (ROS) could accumulate in the cells, and ultimately lead to apoptotic death^27^. In addition, CG8005 gene is a dominant expression gene in the testes of *Drosophila*, and has been documented to be one of the regulatory factors in *Drosophila* testicular reproductive stem cells^20^. Accumulating evidence reported that CG8005, a potential deoxythreonate synthase, is participated in the cell cycle, messenger RNA decay and stress response, and is also closely related to redox homeostasis. However, there are few related research about the biological function and regulatory mechanism of CG8005 gene in *Drosophila* S2 embryonic cells.

In the current study, knockdown of CG8005 induced a number of ROS production, which were then eliminated by the treatment of NAC intervention, suggesting that CG8005 may regulate the oxidative stress in *Drosophila* S2 embryonic cells. In the previous report of professor Yu et al*.*^20,21^ the CG8005 was identified as a regulator of stem cell niche homeostasis in Drosophila testes, and the knockdown of CG8005 could effectively increase ROS concentration in S2 cells, which was similar to the finding of our experiments. It is well known that ROS play a critical role in biological process; whereas high concentration of ROS could cause oxidative damage to cellular biomolecules, thereby contributing to cell death, such as cell apoptosis^28^. NAC, a commonly antioxidant scavenger, was usually used to inhibit intracellular ROS generation to increase the cell survival, which was also similar to the previous report^29^.

## Conclusions

The CG8005 gene in *Drosophila* S2 embryonic cells could regulate the proliferation and apoptosis of S2 embryonic cells by regulate the redox homeostasis, and antioxidant NAC could inhibit cell apoptosis and promote cell proliferation by scavenging ROS in *Drosophila* S2 embryonic cells, which is expected to provide novel insights for the pathogenesis of male infertility and spermatogenesis.

## Data Availability

All data generated or analyzed during this study are included in this published article.
